# Multimodal assessment of the spatial correspondence between fNIRS and fMRI hemodynamic responses in motor tasks

**DOI:** 10.1038/s41598-023-29123-9

**Published:** 2023-02-08

**Authors:** João Pereira, Bruno Direito, Michael Lührs, Miguel Castelo-Branco, Teresa Sousa

**Affiliations:** 1 CIBIT – Coimbra Institute for Biomedical Imaging and Translational Research, University of Coimbra, Coimbra, Portugal; 2 IATV – Instituto do Ambiente, Tecnologia e Vida, University of Coimbra, Coimbra, Portugal; 3 ICNAS – Institute for Nuclear Sciences Applied to Health, University of Coimbra, Coimbra, Portugal; 4 Department of Cognitive Neuroscience, Maastricht Brain Imaging Center, Maastricht University, Maastricht, The Netherlands; 5 Research Department, Brain Innovation B.V., Maastricht, The Netherlands; 6 FMUC – Faculty of Medicine, University of Coimbra, Coimbra, Portugal

**Keywords:** Neuronal physiology, Neurophysiology

## Abstract

Functional near-infrared spectroscopy (fNIRS) provides a cost-efficient and portable alternative to functional magnetic resonance imaging (fMRI) for assessing cortical activity changes based on hemodynamic signals. The spatial and temporal underpinnings of the fMRI blood-oxygen-level-dependent (BOLD) signal and corresponding fNIRS concentration of oxygenated (HbO), deoxygenated (HbR), and total hemoglobin (HbT) measurements are still not completely clear. We aim to analyze the spatial correspondence between these hemodynamic signals, in motor-network regions. To this end, we acquired asynchronous fMRI and fNIRS recordings from 9 healthy participants while performing motor imagery and execution. Using this multimodal approach, we investigated the ability to identify motor-related activation clusters in fMRI data using subject-specific fNIRS-based cortical signals as predictors of interest. Group-level activation was found in fMRI data modeled from corresponding fNIRS measurements, with significant peak activation found overlapping the individually-defined primary and premotor motor cortices, for all chromophores. No statistically significant differences were observed in multimodal spatial correspondence between HbO, HbR, and HbT, for both tasks. This suggests the possibility of translating neuronal information from fMRI into an fNIRS motor-coverage setup with high spatial correspondence using both oxy and deoxyhemoglobin data, with the inherent benefits of translating fMRI paradigms to fNIRS in cognitive and clinical neuroscience.

## Introduction

Functional near-infrared spectroscopy (fNIRS) has emerged as a neuroimaging alternative to measure the hemodynamic cortical brain response and indirectly quantify local changes in neuronal activity^1^. When compared to functional magnetic resonance imaging (fMRI), the gold standard for modern functional neuroimaging, fNIRS increased portability, cost efficiency, temporal resolution, and tolerability to motion artifacts have motivated its popularity in cognitive neuroscience and clinical research^2^.

Despite lower spatial resolution and the inability to perform whole-brain coverage, the aforementioned benefits, in addition to the recent technological and methodological advances in the fNIRS field^3,4^, open the possibility for neuroimaging research in clinical populations and in unconstrained environments that would be highly impractical or not feasible within an MRI setting^5^. Therefore, there is a clear potential upside to understanding the correspondence between these two modalities and how they could be used to complement one another in conditions where fMRI appears less valid^5^.

fMRI relies on the blood oxygen level-dependent (BOLD) contrast, which reflects differences in magnetic susceptibility depending on the concentration of oxygenated and deoxygenated blood driven by localized changes in brain blood flow and oxygenation consumption. Such differences are coupled to underlying neuronal activity based on the mechanism of neurovascular coupling response^6^. In turn, fNIRS relies on the emission of light with different wavelengths within the near-infrared spectrum (650–1000 nm) and the transparency of human tissue, such as the brain tissue to it^7^. The measurement of the differential neuronal tissue light absorption rate of oxygenated and deoxygenated blood (oxy- and deoxyhemoglobin: HbO and HbR) allows for monitoring hemodynamic changes linked with evoked brain activity^8^.

Considering the balloon model^9^ and the interplay between the cerebral metabolic rate of oxygen (CMRO_2_), cerebral blood volume (CBV), and flow (CBF), a theoretical relation can be drawn between the relative changes in BOLD response to changes in concentration levels of HbR. In a review of concurrent fMRI-fNIRS studies, Steinbrink et al.^10^ concluded that temporal correlation between the BOLD contrast and changes in HbR concentration was considered the common denominator between the two modalities although not solely dependent on the concentration of HbR, with studies reporting higher temporal correspondence with total hemoglobin (HbT)^11^, and with a similar level between HbO and HbR^12^.

More recently, an overview by Scarapicchia et al.^5^ reported a wide variety of multimodal fMRI-fNIRS imaging studies, including motor^13–16^, visual^17,18^, language processing^19,20^, working memory tasks^21–23^, and resting-state networks^24,25^ showing that both HbO and HbR can detect hemodynamic changes due to spontaneous and task-related cortical activation. From these studies, HbO was reported to have an overall higher temporal correlation^13^ with the fMRI BOLD signal, despite the wide variance of the correlation (ranging from 0 to 0.8). Additionally, both high levels of temporal correlation between amplitudes of the fMRI BOLD signal and HbO (r = 0.65) and HbR (r = − 0.76)^22,23^, as well as mean correlation as low as |r| ∼ 0.2 were reported^26^. In addition, analysis of the spatial correspondence between fMRI and fNIRS measurements has been less extensively studied, beyond qualitative comparisons, with Huppert et al.^16^ reporting good correspondence between fMRI and fNIRS signals, with higher levels of spatial cortical correlation with HbO, through an image reconstruction method based on cortical surface topology, with overall results highly impacted by lower sensitivity in subcortical areas.

Taking this into account, here, we aimed to add insight into the spatial interplay between these hemodynamic signals using a novel multimodal approach. Subject-specific fNIRS data (HbO, HbR, and HbT) were used to model asynchronously recorded fMRI during a motor-task paradigm. We hypothesized that it is possible to use fNIRS-based cortical signals from motor-related regions to identify corresponding brain regions in previously acquired fMRI data. Furthermore, we hypothesize that different chromophores provide different information, and we aim to determine which type of fNIRS measurement is able to transfer a greater amount of spatial neuronal activity information concerning the corresponding fMRI BOLD response. This was tested by comparing activation in primary motor cortices (M1) and premotor cortices (PMC) on fMRI data modeled by the different fNIRS measurements.

## Methods

### Participants

Nine volunteers (mean age: 28.5 ± 3.3; 2 female) with no history of neurological or psychiatric diseases were recruited for asynchronous fMRI and fNIRS data acquisition. All had normal or corrected-to-normal vision and no history of neurological or psychiatric diseases. All volunteers gave informed consent before participating. This study was conducted following the declaration of Helsinki. This project was approved by the Ethics Committee of the University of Coimbra—CE_Proc. CE-061/2021.

### Experimental paradigm and instructions

For this experiment, we replicated the experimental design of the first functional run of a previous fMRI experiment^27^, which combined motor imagery with correspondent actual motor performance. The functional run included two activation conditions, motor action (MA) and motor imagery (MI), and Baseline periods in a block design experiment. The mapping procedure comprised a total of 17 blocks with a duration of 30 s—9 Baseline blocks, 4 MA blocks, and 4 MI blocks. The total duration of the run was 8 min and 30 s. At the beginning of each block, the condition name was presented to the participant on the screen for 2 s. During the MA blocks, each participant was instructed to execute a bilateral finger tapping sequence: 1-2-1-4-3-4 (1—left middle finger, 2—left index, 3—right index, and 4—right middle finger) at a specific frequency (p.e, 2 Hz, demonstrated before data acquisition). During the MI blocks, the participants were asked to imagine the same sequence, without overt movement.

### Data acquisition

#### fMRI acquisition parameters description

The setup includes a 3 T Siemens Magnetom TimTrio (Siemens, Erlangen, Germany) scanner with a 12-channel head coil. First, the participants underwent a high-resolution magnetization-prepared rapid acquisition gradient echo sequence for coregistration of functional data (176 slices; echo time [TE]: 3.42 ms; repetition time [TR]: 2530 ms; voxel size: 1 × 1 × 1 mm; flip angle [FA]: 7°; field of view [FOV]: 256 × 256 mm). Functional imaging focused on motor-related areas (related to motor planning and/or execution in frontal and parietal areas). The acquisition consisted of an echo-planar imaging sequence with 26 slices, in-plane resolution: 3 × 3 mm, FOV: 210 × 210 mm, slice thickness: 3.5 mm, FA: 75°, TR = 1500 ms, and TE = 30 ms.

#### fNIRS setup and acquisition parameters description

**Figure 1:**
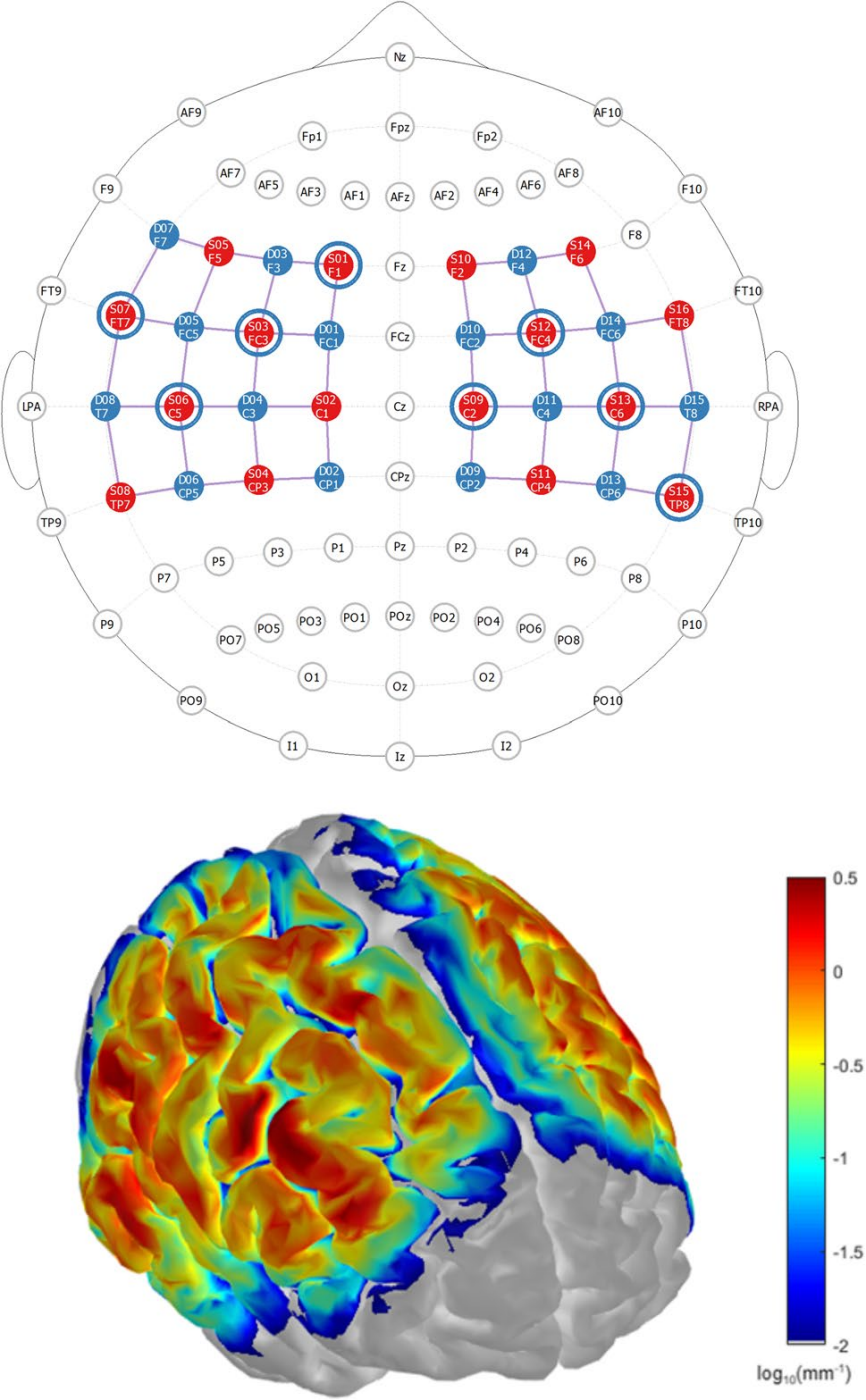
Probe design and spatial sensitivity. Top: Two dimension visualization of the optode array with 16 sources (red dots) and 15 detectors (blue dots) resulting in 54 channels covering bilateral sensorimotor areas. Blue circles represent the location of SSD evenly distributed throughout the montage. Bottom: Cortical spatial sensitivity profile of the montage in log10(mm^−1^) calculated using a Monte Carlo simulation including 10^06^ photons calculated and visualized using Atlasviewer^28^.

**Figure 2:**
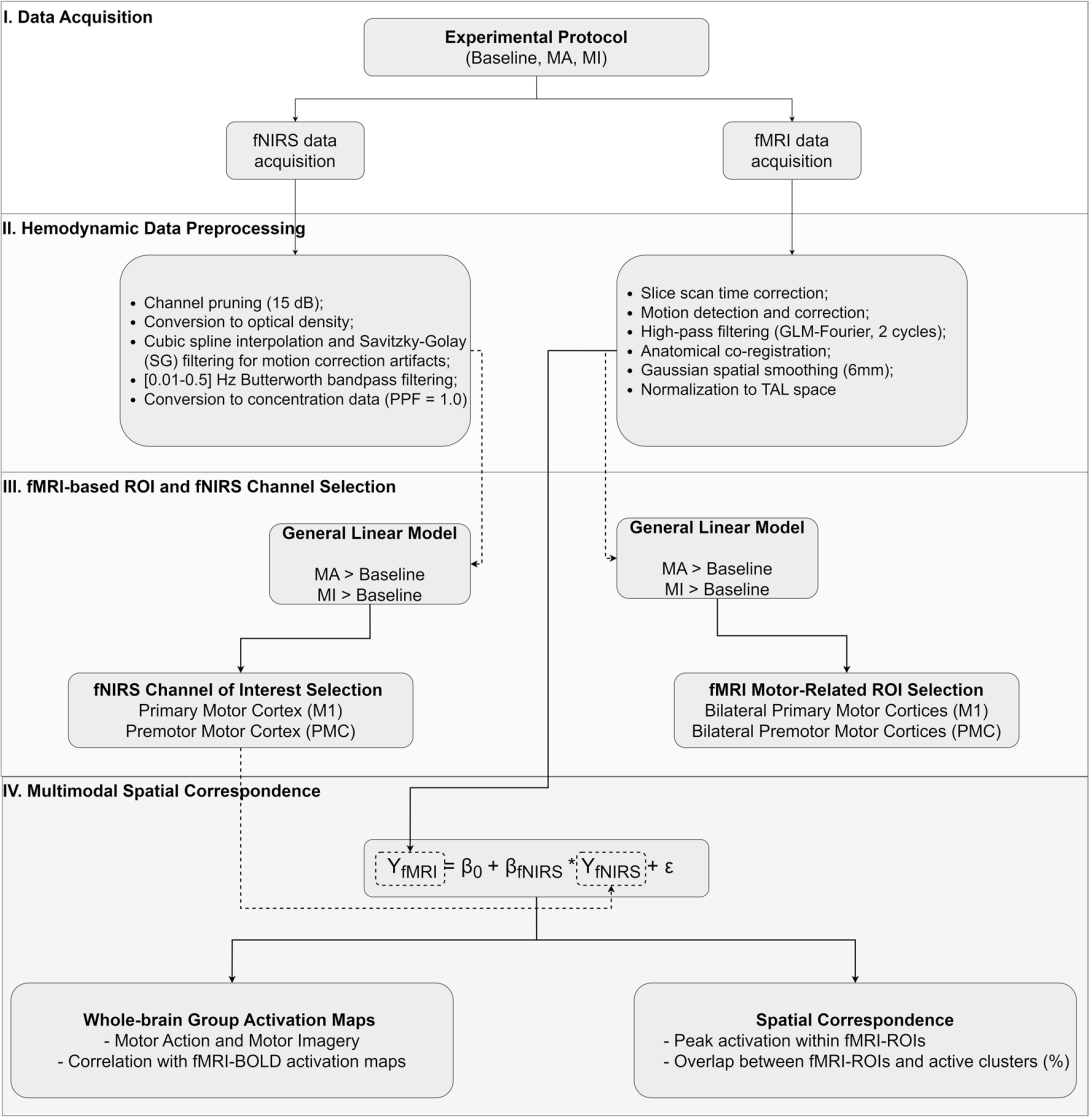
Overview of the multimodal methodological approach. Asynchronous fNIRS and fMRI data acquired using the same experimental protocol (**I**) were preprocessed for each subject (**II**). After fNIRS preprocessing, the resulting oxy, deoxy, and total-hemoglobin time courses were selected based on the GLM analysis for the contrasts MA > Baseline and MI > Baseline (**III**). These concentration timecourses were integrated as a single GLM predictor into the fully preprocessed fMRI data (**IV**). From the resulting subject-level activation maps, the peak *t*-value and proportion of voxels overlapping with the individual ROIs corresponding to the bilateral motor and premotor cortices served as spatial correspondence metrics (**IV**). Group-level whole-brain activation maps analysis was performed using the individually selected channels for both contrasts (**IV**).

The hemodynamic cortical response was assessed using the portable NIRSport2 continuous wave (CW) fNIRS system (Nirx Medical Technologies, Berlin), sampled at 5.08 Hz and using Aurora v2021.1 acquisition software. The experimental setup consisted of 16 LED light sources (λ1 = 760 nm; λ2 = 850 nm) and 15 Silicon Photodiode (SiPD) detectors, with an intra-optode distance of 30 mm, covering bilateral motor areas, using a total of 54 channels (Fig. 1). To mitigate potential extracerebral confounds, eight additional short-distance detectors (SDD) (8 mm) were evenly distributed throughout the setup (Fig. 2I).

### Hemodynamic data preprocessing

#### fMRI preprocessing

Offline fMRI data analysis was performed using BrainVoyager QX 2.8 (Brain Innovation, the Netherlands) (Fig. 2II). Preprocessing included slice scan time correction, motion detection and correction, temporal high-pass filtering (GLM-Fourier, 2 cycles), co-registration between the functional and anatomical data, spatial smoothing with a Gaussian filter (FWHM = 6 mm), and normalization to Talairach (TAL) coordinate space. Single-subject functional data was modeled using a General Linear Model (GLM). The design matrix was determined based on the predictors encoding the stimulus conditions, combined with six motion parameters (three translational and three rotational) and spikes (i.e., outliers in the BOLD time course), which were included as covariates. For each subject, individual regions of interest (ROIs) corresponding to left and right primary motor cortices (lM1 and rM1) were selected based on the activation clusters for the contrast analysis (MA > Baseline) and left and right premotor cortices (lPMC, rPMC) were selected based on the activation clusters for the contrast analysis (MI > Baseline). ROI selection for M1 was performed with the false discovery rate (FDR) correction method (q_FDR_ < 0.005) and (q_FDR_ < 0.05) for the PMC, to address multiple comparison limitations, and take into account anatomical landmarks (Fig. 2III).

#### fNIRS preprocessing

fNIRS data preprocessing was performed in MATLAB R2021a (Mathworks, USA) using the Homer3 v1.33.0^29^ (Fig. 2II). Channels with insufficient raw data quality (signal to noise ratio (SNR), calculated using the quotient between the mean of the raw signal by its standard deviation, lower than 15 dB) were pruned. Raw intensity signals were converted to changes in optical density calculated from the normalized changes in light intensity incident in the detector from its paired source position^29^. Variations over 5 optical density units throughout half a second were identified as motion-related artifacts and were corrected using cubic spline interpolation with Savitzky-Golay (SG) filtering^30^. A Butterworth bandpass filter (3rd-order lowpass and 5th-order highpass) with cut-off frequencies of 0.01–0.5 Hz was applied to remove low-frequency drifts as well as part of non-hemodynamic related signal components such as heart rate. Data were then converted to variations of concentration of oxy- and deoxygenated hemoglobin (HbO and HbR) using the modified Beer-Lambert Law, incorporating a partial pathlength factor (PPF) of 1.0 for both wavelengths. A fixed-effects (FFX-GLM) group analysis was performed using Satori 1.6 (Brain Innovation, the Netherlands)^31^, to determine active cortical areas considering the contrasts MA > Baseline and MI > Baseline condition (*N* = 9, Bonferroni corrected, *q* < 0.05).

#### fNIRS channel selection and data extraction

To determine the fNIRS channels of interest for each participant we calculated a GLM. The hemodynamic response function (HRF) was modeled using a consecutive sequence of Gaussian functions, each with a standard deviation of 1.0 s and temporally spaced 1.0 s apart, as done by Yücel^32^. For each source-detector pair, the influence of systemic physiology and extracerebral contaminations from superficial layers were mitigated by performing short separation regression (SSR)^32^ using as a regressor the data from the most correlated short channel^33^. The beta values (weight of regressors) were obtained using ordinary least squares fit. For each subject, HbO, HbR, and HbT (total hemoglobin: HbT = HbO + HbR) time courses of concentration data of selected channels were extracted. The criterion for the subject-level channel selection was based on the highest t-value for the contrast analysis (MA > Baseline) for channels covering areas more active during overt movement, and (MI > Baseline) for channels more linked with covert movement (Fig. 2III). As a control measure, for each subject, a channel unrelated to the motor task (i.e., the channel with a t-value nearest to null for the MA > Baseline) was selected for data extraction.

To ensure the fNIRS data quality of the selected channels, the coefficient of variation (CV) was calculated for the raw data as in Piper et al.^34^. To avoid errors arising from assumptions in response functions, the preprocessing pipeline for the fNIRS raw data of the selected channels was repeated for the multimodal analysis, including the same preprocessing steps as described earlier and the SSR, independent of any model definition. A frequency matching factor (TR_fMRI_ × f_fNIRS_) was applied to the preprocessed fNIRS data to downsample its temporal frequency to match the TR of the acquired fMRI data. This, in addition to the replication of the experimental protocol, assured equal and matching sampling points (N = 340) for both modalities. Block-related averaging (i.e., an average of the response for all the participants, measured as a percent signal change % within a condition block) hemodynamic responses of both modalities were included for comparison.

### Multimodal spatial correspondence

Subject-specific multimodal integration of hemodynamic data was done by performing a GLM on the fMRI data in which the design matrix consisted of a single predictor containing the subject’s corresponding fNIRS time course previously selected as follows:

1 $${Y}_{fMRI}={\beta }_{0}+ {\beta }_{fNIRS}\times {Y}_{fNIRS}+ \varepsilon ,$$

where ${Y}_{fMRI}$ is the measured time course of an fMRI single voxel and ${Y}_{fNIRS}$ is the measured variation of fNIRS signal (HbO, HbR, or HbT) of the selected channel of interest and serves as a reference function (i.e., predictor), ${\beta }_{fNIRS}$ being the weight of its contribution in explaining the variance of ${Y}_{fMRI}$, ${\beta }_{0}$ a constant representing the weight of a baseline signal, and an error value of $\varepsilon$ (Fig. 2IV). For each subject and corresponding fMRI run data, the time courses of concentration data of HbO, HbR, and HbT were used. Firstly, a whole-brain fixed effects (FFX-GLM) group analysis was performed, combining all predictors representing the selected channel for all subjects, per chromophore. Group-level multimodal spatial correspondence was accessed for ‘Motor Action’ (i.e., using as predictor channels selected based on the MA > Baseline), repeated for ‘Motor Imagery’, (i.e., with channels selected based on the MI > Baseline), and controlled using channels unrelated with motor tasks.

This was done to inspect the ability to identify motor-related regions’ activation clusters in fMRI data using only fNIRS-based cortical signals as predictors of interest. Group-level activation map spatial specificity was inspected as in Lührs et al.^35^, using Spearman’s Correlation on the distribution of the t statistics of all voxels depicting the brain signal of each group-level map. All combinations (activation maps from fMRI data for contrasts MA > Baseline and MI > Baseline, maps from fMRI data modeled using as regressor HbO, HbR, and HbT concentration data—using selected channels based on the contrast MA > Baseline, MI > Baseline, and unrelated with the task) were reported with correction for multiple comparisons. Individual-level spatial correspondence between fMRI and fNIRS data was analyzed based on the activation clusters of the subject-specific GLM analysis and the location of the individually selected ROIs. Individual-level multimodal spatial correspondence was analyzed for ‘Motor Action’ (i.e., using the selected fNIRS channel for the MA > Baseline contrast and the fMRI selected M1 ROI) and ‘Motor Imagery’ (i.e., using the selected fNIRS channel based on the MI > Baseline contrast and the fMRI selected PMC ROI). For each ROI, the absolute peak t-value of the activated cluster, as well as the proportion of statistically significant voxels (considering p < 0.05 for the contrast of interest) were extracted. Possible differences in spatial correspondence between the different fNIRS chromophores (HbO, HbR, and HbT) and the BOLD signal were assessed by computing a one-way analysis of variance (ANOVA) on these output metrics.

## Results

### fMRI motor-related areas and fNIRS channels of interest selection

**Figure 3:**
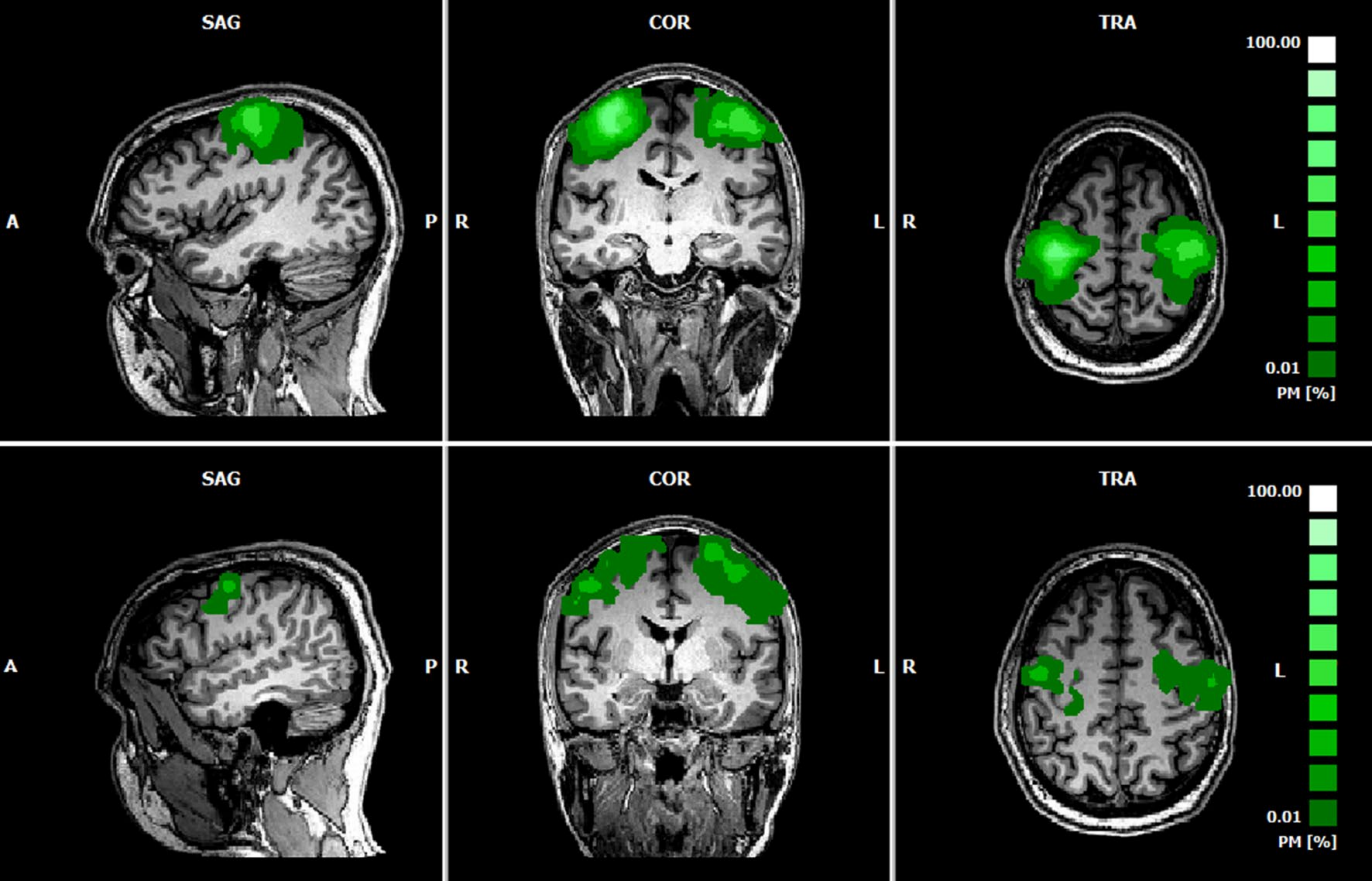
Probabilistic map of the combined ROIs (top: lM1 and rM1; bottom: lPMC and rPMC) for the 9 subjects, highlighting the overlap of the subject-specific ROIS.

For each subject, significant activation clusters were selected as M1and PMC ROIs, based on fMRI data. The mean location of the M1 ROIs were mean_lM1_: (− 36, − 18, 55) TAL coordinates, with size 6179 ± 2152 mm^3^ and mean_rM1_: (34, − 21, 56), with size 7581 ± 2337 mm^3^ (MA > Baseline contrast, q_FDR_ < 0.005). The mean location of the PMC ROIS were mean_lPMC_: (− 40, − 7, 52) TAL coordinates, with size 3817 ± 2372 mm^3^ and mean_rPMC_: (34, − 7, 54), with size 2479 ± 2774 mm^3^ (MI > Baseline contrast, q_FDR_ < 0.05). The probabilistic map corresponding to the overlap of the subject-specific ROIs is presented in Fig. 3.

**Figure 4:**
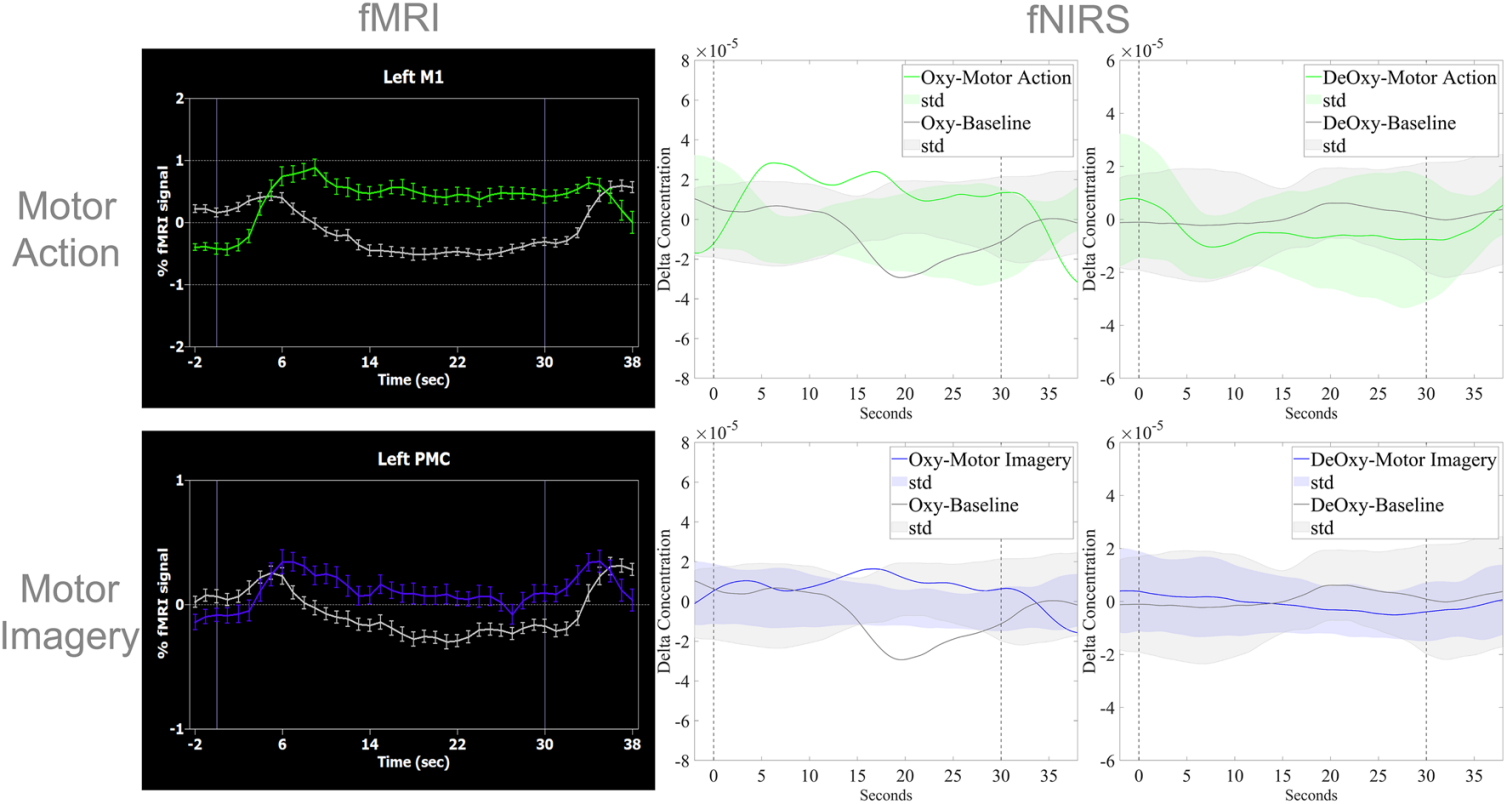
Group fMRI and fNIRS hemodynamic response during motor action and motor imagery. Top: Average response for all participants, measured as a percent signal change (PSC) for MA (green) and Baseline (grey) based on fMRI data corresponding to the left M1 (Bonferroni corrected, *q* < 0.05), and concentration data of an fNIRS channel corresponding to left M1 (Bonferroni corrected, *q* < 0.05) of HbO and HbR. Bottom: Average response for all participants, measured as a PSC for MI (blue) and Baseline (grey) of fMRI data corresponding to the left PMC (Bonferroni corrected, *q* < 0.05), and concentration data of an fNIRS channel corresponding to the left PMC (Bonferroni corrected, *q* < 0.05) of HbO and HbR. In the fMRI block-related averaging, variation bars correspond to the standard error and in the fNIRS block-related averaging, the shaded area corresponds to the standard deviation.

The fNIRS channels covering brain regions more activated during bilateral MA and MI were also selected, one per chromophore and per subject (HbO, HbR, and HbT). Take note that the HbT channel chosen is the same as for the HbO. Related with overt movement, the pair source-detector selected, as well as the t-value from the contrast analysis MA > Baseline and the corresponding TAL coordinates, are presented in Table 1. Regarding imagined movement, the pair source-detector selected, as well as the t-value from the contrast analysis (MI > Baseline) and the corresponding TAL coordinates, are presented in Table 2. The mean location of the channels selected was calculated by projecting the probe onto the cortex using Atlasviewer^28^ and using a nonlinear registration from MNI to TAL coordinates^36^. The coordinates were mean_lM1_HbO_: (− 38, 2, 40); mean_rM1_HbO_: (36, 0, 43); mean_lM1_HbR_: (− 42, − 22, − 40); mean_rM1_HbR_: (40, − 13, 42) and mean_lPMC_HbO_: (− 36, 12, 26); mean_rPMC_HbO_: (36, 28, 19); mean_lPMC_HbR_: (− 28, 3, 25); and mean_rPMC_HbR_: (36, − 25, 46) TAL. All but one mean location of a channel selected based on the contrast MA > Baseline, mean_lM1_HbR_, was within the fMRI M1 cluster probabilistic map previously presented (Fig. 4) The same correspondence was not achieved with channel mean location for channels based on the contrast MI > Baseline and the PMC probabilistic map. Raw data CV of selected channels was mean_CV_HbO_ = 1.73 ± 0.65 for the HbO and of the HbR channels was mean_CV_HbR_ = 2.32 ± 1.02.

**Table 1:** Selected channels for fNIRS data extraction for all subjects, per chromophore, based on the t statistics for MA > Baseline contrast analysis (uncorrected) and its corresponding TAL coordinates, calculated by projecting the probe onto the cortex using Atlasviewer^28^ and using a nonlinear registration from MNI to TAL coordinates^36^.

Chromophore	Channel	Subjects	t statistics (MA > Baseline)	TAL coordinates (Hemisphere)
HbO	S9-D11	2	30.63; 25.02	(35 − 16 45) (R)
	S2-D2	1	20.07	(− 25 32 26) (L)
	S3-D1	1	15.61	(− 36 12 49) (L)
	S3-D4	1	16.41	(− 42 − 6 39) (L)
	S4-D4	1	27.34	(− 48 − 27 45) (R)
	S9-D10	1	14.53	(28 − 2 51) (R)
	S10-D12	1	26.94	(29 40 31) (R)
	S12-D11	1	15.58	(52 − 5 43) (R)
HbR	S11-D11	2	− 31.57; − 24.81	(45 − 29 46) (R)
	S2-D4	1	− 27.69	(− 39 − 16 54) (L)
	S3-D5	1	− 23.56	(− 46 8 26) (L)
	S4-D2	1	− 29.72	(− 40 − 40 58) (L)
	S4-D4	1	− 17.37	(− 48 − 27 45) (L)
	S6-D8	1	− 31.56	(− 37 − 33 17) (L)
	S9-D9	1	− 29.62	(31 − 27 61) (R)
	S14-D12	1	− 23.76	(38 33 15) (R)

**Table 2:** Selected channels for fNIRS data extraction for all subjects, per chromophore, based on the t statistics for MI > Baseline contrast analysis (uncorrected) and its corresponding TAL coordinates, calculated by projecting the probe onto the cortex using Atlasviewer^28^ and using a nonlinear registration from MNI to TAL coordinates^36^.

Chromophore	Channel	Subjects	t statistics (MI > Baseline)	TAL coordinates (Hemisphere)
HbO	S1-D1	1	18.67	(− 18 34 53) (L)
	S3-D5	1	24.45	(− 35 11 26) (L)
	S4-D2	1	13.30	(− 32 − 39 53) (L)
	S4-D4	1	22.03	(− 42 − 25 44) (L)
	S5-D5	1	14.32	(− 34 22 21) (L)
	S7-D7	1	16.15	(− 44 19 1) (L)
	S8-D6	1	19.43	(− 62 − 41 13) (L)
	S11-D11	1	29.21	(36 − 25 46) (R)
	S12-D14	1	22.27	(61 21 35) (R)
HbR	S5-D7	2	− 25.44; − 15.80	(− 29, 38, 8) (L)
	S11-D11	2	− 25.98; − 11.70	(36 − 22 42) (R)
	S2-D1	1	− 21.42	(− 24 2 58) (L)
	S3-D3	1	− 11.85	(− 37 26 33) (L)
	S4-D2	1	− 29.74	(− 32 − 39 53) (L)
	S4-D4	1	− 19.05	(− 42 − 25 44) (L)
	S6-D8	1	− 24.70	(− 56 − 14 11) (L)

Block-related averaging of the hemodynamic responses for both modalities is presented in Fig. 4, showing the expected increase in fMRI signal in voxels corresponding to LM1 and the corresponding increase in HbO/decrease in HbR signals in a channel corresponding to LM1 during MA (green), and similar behavior for MI in voxels/channel corresponding to LPMC during MI (blue), both controlled for the Baseline condition (grey).

### Multimodal fNIRS/fMRI spatial correspondence

#### Whole-brain group-level analysis

**Figure 5:**
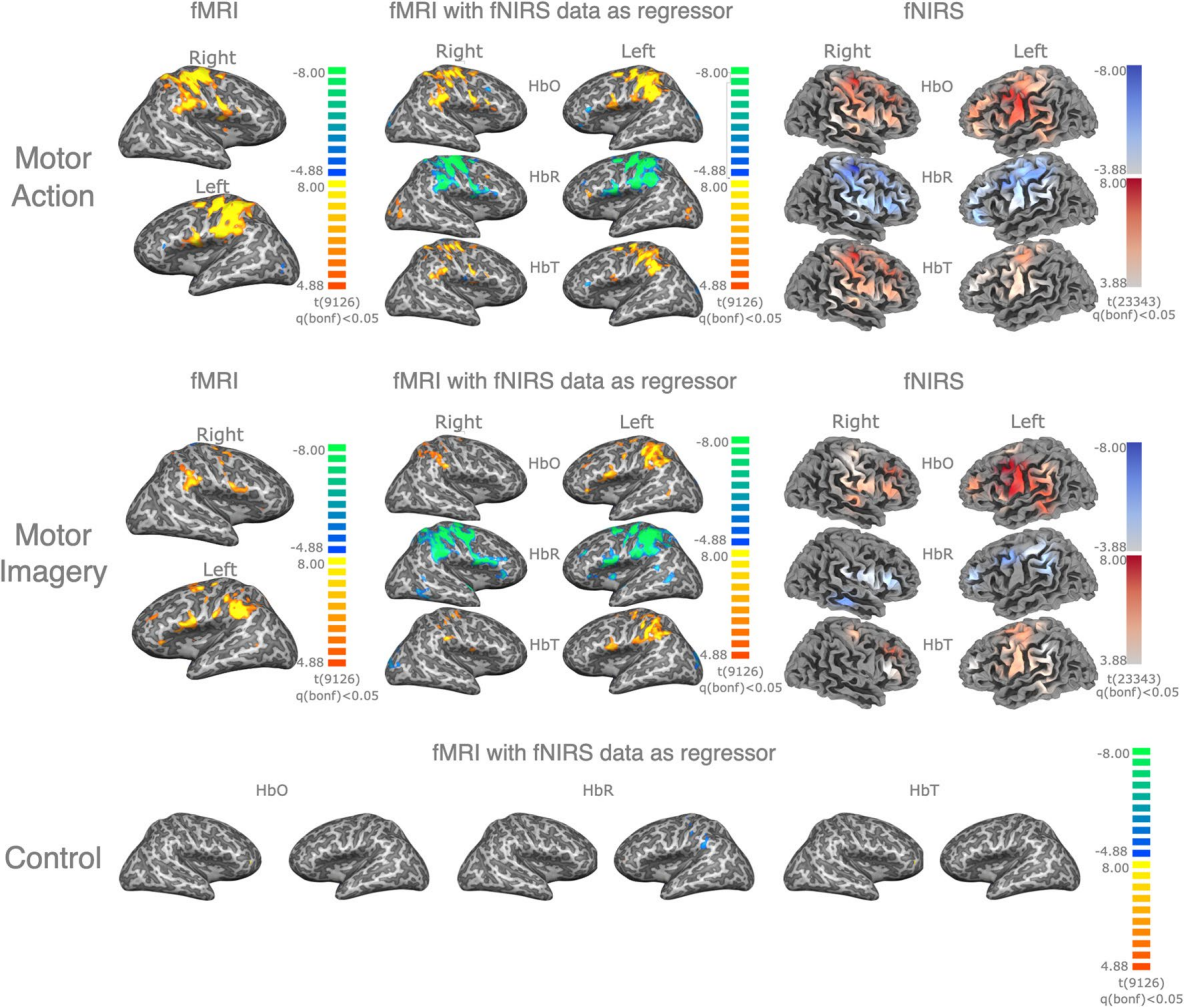
Whole-brain group-level analysis. Top row: Group-level analysis for ‘Motor Action’. Left column—Group FFX-GLM fMRI activation map for both right and left hemispheres for the contrast MA > Baseline (*N* = 9, Bonferroni corrected, *q* < 0.05). Centre column—Multimodal group FFX-GLM activation maps (Bonferroni corrected, *q* < 0.05) of fMRI data modeled using as regressor extracted fNIRS data (N = 9 channels, single-channel per subject) from each fNIRS measurement (HbO, HbR, and HbT), selected based on the contrast MA > Baseline. Right column—Group-level FFX-GLM fNIRS activation map for each fNIRS measurement (HbO, HbR, and HbT) based on the same contrast (N = 9, Bonferroni corrected, *q* < 0.05). Middle row: Group-level analysis for ‘Motor Imagery’. Left column—Group FFX-GLM fMRI activation map for both right and left hemispheres for the contrast MI > Baseline (N = 9, Bonferroni corrected, *q* < 0.05). Centre column—Multimodal group FFX-GLM activation maps (Bonferroni corrected, q < 0.05) of fMRI data modeled using as regressor extracted fNIRS data (N = 9 channels, single-channel per subject) from each fNIRS measurement (HbO, HbR, and HbT), selected based on the contrast MI > Baseline. Right column—Group-level FFX-GLM fNIRS activation map for each fNIRS measurement (HbO, HbR, and HbT) based on the same contrast (N = 9, Bonferroni corrected, *q* < 0.05). Bottom row: Control multimodal group FFX-GLM activation maps (Bonferroni corrected, *q* < 0.05) of fMRI data modeled using as regressor extracted fNIRS data unrelated to the motor tasks (N = 9 channels, single-channel per subject) from each fNIRS measurement (HbO, HbR, and HbT).

In Fig. 5 the multimodal fNIRS/fMRI spatial correspondence is presented, with a whole-brain FFX group analysis (*N* = 9; *q* < 0.05, Bonferroni corrected) of activation maps from fMRI data modeled from the individually-selected channels chosen taking into consideration the contrasts MA > Baseline, MI > Baseline and Control (HbO, HbR, and HbT), and the corresponding fMRI group-level activation map (*N* = 9; *q* < 0.05, Bonferroni corrected), and fNIRS group-level activation pattern (*N* = 9; *q* < 0.05, Bonferroni corrected), for the same contrast.

**Figure 6:**
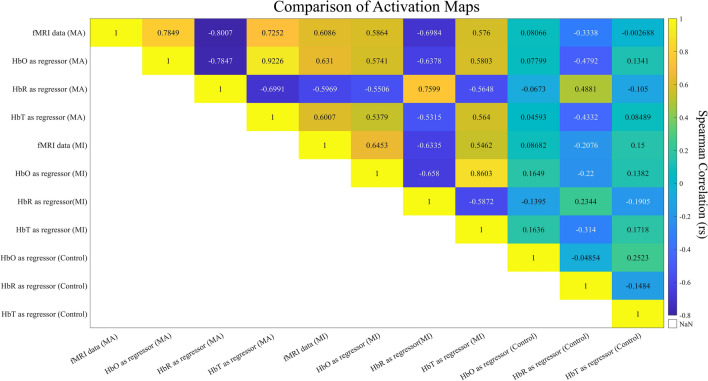
Spatial correlation matrix of group-level brain activation maps. Spearman’s correlation (*rs*) of the distribution of the *t-*statistic of each group activation map (fMRI data for contrasts MA > Baseline and MI > Baseline, fMRI data modeled using as regressor HbO, HbR, and HbT concentration data—using selected channels based on the contrast MA > Baseline, MI > Baseline, and unrelated with task). All combinations except one (‘fMRI data (MA)’, ‘HbT as a regressor (Control)’) were significant at *q* < 0.05, Bonferroni corrected.

Spatial correspondence between group-level activation maps, as well task-related specificity, can be assessed in Fig. 6, with a spatial correlation matrix of the distribution of the t statistics of all voxels depicting the brain signal of each map for all group-level activation maps. High absolute levels of correlation were found between fMRI data and fMRI data modeled from fNIRS channels, accounting for both MA > Baseline and MI > Baseline, as well as for all chromophores, with p < 0.05 corrected for multiple comparisons. The same behavior was not found when comparing fMRI data with fMRI data modeled from channels unrelated to the task (Control).

**Figure 7:**
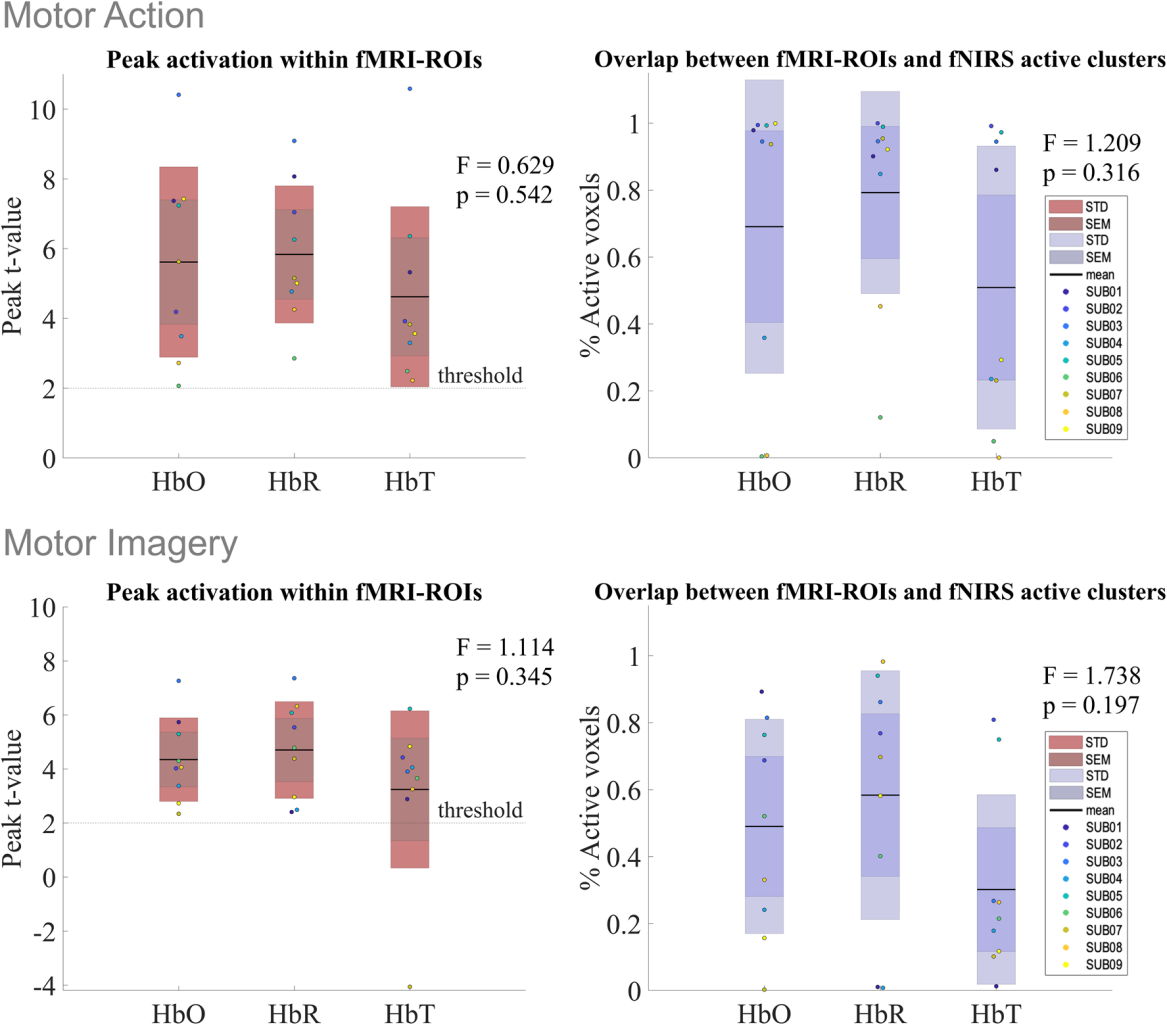
Color-coded subject-level fNIRS/fMRI spatial correspondence. Top: Subject-level analysis for ‘Motor Action’. Left—distribution of the peak t-value of the activation clusters. The dotted line represents the threshold of activation (i.e., t-value greater than 1.96 resulting from the GLM using fNIRS measurements—HbO, HbR, and HbT). Right—the proportion of active voxels (t-value greater than 1.96) from the single selected channel per subject within the fMRI-based M1. Bottom: Subject-level analysis for ‘Motor Imagery’. Left—distribution of the peak t-value of the activation clusters. The dotted line represents the threshold of activation (i.e., t-value greater than 1.96 resulting from the GLM using fNIRS measurements—HbO, HbR, and HbT). Right—the proportion of active voxels (t-value greater than 1.96) from the single selected channel per subject within the fMRI-based PMC. The black line corresponds to the mean value across chromophores, the darker-colored area represents the standard error of the mean (mean ± SEM), and the lighter color corresponds to the standard deviation (mean ± SD) for each output (red: peak t-value; purple: proportion of active voxels).

The subject-level spatial correspondence between fMRI data modeled from selected fNIRS measurements and corresponding fMRI measured BOLD signal within the individually selected ROI (M1 for MA > Baseline; PMC for MI > Baseline) corrected for the hemisphere of the chosen channel is presented in Fig. 7, where peak t-value from active clusters and proportion of active voxels within the selected ROIs coordinates is shown for HbO, HbR, and HbT, for both contrasts. Regarding ‘Motor Action’ (MA > Baseline contrast), mean peak activation, per chromophore was [mean_HbO_ = 5.62 ± 2.73; mean_HbR_ = 5.84 ± 1.94; mean_HbT_ = 4.62 ± 2.58] with a proportion of active voxels of [overlap_HbO_ = 0.69 ± 0.44; overlap_HbR_ = 0.79 ± 0.30; overlap_HbT_ = 0.51 ± 0.42]. For ‘Motor Imagery’ (MI > Baseline contrast), mean peak activation, per chromophore was [mean_HbO_ = 4.35 ± 1.55; mean_HbR_ = 4.70 ± 1.79; mean_HbT_ = 3.24 ± 2.91] with a proportion of active voxels of [overlap_HbO_ = 0.40 ± 0.32; overlap_HbR_ = 0.58 ± 0.37; overlap_HbT_ = 0.30 ± 0.28]. For both contrasts, there was no significant effect on the multimodal spatial correspondence depending on the type of fNIRS signal used for the peak activation [F_MA_(2,9) = 0.629, p_MA_ = 0.542; F_MI_(2,9) = 1.114, p_MI_ = 0.345] nor for the proportion of active voxels [F_MA_(2,9) = 1.209, p_MA_ = 0.316; F_MI_(2,9) = 1.738, p_MI_ = 0.197].

Inter-subject variability on the multimodal fNIRS/fMRI spatial correspondence, illustrated in Fig. 5, is noticeable in peak t-values across fNIRS measurements, ranging from highly activated to borderline statistically non-significant, for both ‘Motor Action’ and ‘Motor Imagery’. Regarding ‘Motor Action’, higher peak activation was more reported using HbR data (N = 5) and lower peak activation was reported using HbT data (N = 5). In most subjects, there is a clear overlap between active clusters and individually defined M1, across chromophores, with percentages of active clusters above 80%. Overlap values lower than 40% are reported for HbO (N = 2) and HbR (N = 1) but this behavior is more prevalent in HbT (N = 5). Subjects who show lower overlap values across chromophores (SUB06, SUB08) also present lower values of peak activation. For the ‘Motor Imagery’ analysis, higher peak activation was more reported using HbR data as a regressor (N = 6) and lower when using HbO (N = 5), with a reported inversion of expected signal in activation in one participant using HbT. Overlap between active clusters and individually defined PMC is present in most subjects, although lower when compared with the ‘Motor Action’ analysis, with most of the overlap values for HbT below 40%.

## Discussion

The main contribution of this work was the analysis of the spatial correspondence between the BOLD signal and corresponding fNIRS measurements using a motor paradigm in healthy participants, with a novel multimodal approach. We hypothesized that modeling fMRI data using as regressors its corresponding fNIRS measurements (HbO, HbR, and HbT) from the same subject, acquired asynchronously with the same experimental protocol, would allow for the identification of active clusters corresponding to motor-related areas.

### Selection of regions and channels of interest

fMRI-based ROI selection is validated through the high overlap values in the probabilistic maps (Fig. 4), with lM1 and rM1voxels presenting values reaching 100% in both ROIs in a normalized space, and lPMC and rPMC reaching 60–80%, meaning that they were contained in all or most subject-level chosen ROIs, respectively.

For the fNIRS channel selection, several channels were selected as of interest, varying in hemisphere and location in the brain, for both contrasts. Nonetheless, a match between mean coordinates of fNIRS channels and fMRI ROIs probabilistic maps for the contrast MA > Baseline is reported in all combinations (chromophore, hemisphere) but one, covering more anterior regions. The same was not reported for the MI > Baseline, nonetheless the fact that the source and detector placement was done using the international 10/5 system and the correct definition of the reference Cz provides some reassurance on the correct position of the probe for the channel selection^37^. However, no digitalization of optodes position was performed on the subject-level, limiting our ability to address inter-subject anatomical variability. Importantly, this limitation is mitigated by the fact that channel selection was based on functional data, targeting areas more active in MA or MI, comparing it to during Baseline, and not on anatomical landmarks, as confirmed by the block-related averaging plots and relatively high values of task-related t statistics.

### Multimodal fNIRS/fMRI spatial correspondence

Group-level analysis of fMRI data modeled from fNIRS measurements exhibits significant and consistent activation in motor-related areas, with spatial correspondence with data modeled from corresponding BOLD fMRI response, using a motor paradigm containing both covert and overt movement. Using data from a single fNIRS channel per subject, significant activation in bilateral M1, premotor areas (PMC), and more medial supplementary motor areas (SMA) are reported. These findings are consistent with the motor network involved in motor planning and execution and go in line with what is expected during MA^38^ with similar activation patterns reported in the corresponding fMRI and fNIRS response. This activation appears to be present regardless of the chromophore (HbO, HbR, or HbT) from which the data are modeled, as well as task-related specific, i.e., with higher spatial correlation within the task (fMRI activation pattern with fMRI data modeled from channels selected from the same contrast) and lower when comparing with channels unrelated with motor-tasks. This same pattern was found when comparing subject-level activation clusters within individually defined M1 and PMC, corrected for the hemisphere of the chosen channel. No effect on the multimodal spatial correspondence was found, depending on the fNIRS measurements used for both peak activation metrics.

Our results suggest that most participants present activation clusters overlapping motor-related regions when modeling fMRI data with corresponding fNIRS measurements. However, we found intersubject variability in how fNIRS and its corresponding BOLD responses are intertwined. Additionally, there was no clear pattern on how these results depended on data correspondence to oxy, deoxy, or total-hemoglobin concentration time points. The match between the subject/chromophore combination with subpar results for both peak activation and proportion of active voxels works here as an important validation of the choice of spatial correspondence output metrics.

Ultimately, we aimed at understanding the spatial interplay between measurements from two neuroimaging modalities and possibly determining which fNIRS type of measurement was able to transfer a greater amount of spatial neuronal activity information concerning the corresponding fMRI BOLD response. According to our results, using only a single channel per subject, both oxy and deoxyhemoglobin data allowed us to detect and identify cortical activation. This suggests that it is possible to translate activity information from fMRI motor paradigms into an fNIRS setup guaranteeing proper data quality. However, clear disentanglement of the BOLD contrast was not feasible with this dataset, with group-level activations providing data from all chromophores. This attests to the complexity of the BOLD contrast as it is measured within the magnetic resonance environment. Regional increases in CMRO_2_ and therefore deoxygenated blood due to local activation of neurons are linked with the regional increase of CBF and CBV which creates a remnant surplus of oxygenated blood, suggesting that the BOLD signal relies on local deoxy-hemoglobin content, but partially on its oxygen saturation, especially considering long duration task block^39^.

These findings are in line with several fMRI–fNIRS studies that aim to use one signal to understand the underlying mechanisms or the hemodynamic response of the other^40–43^. Moreover, our findings also contribute to the discussion on the comparison between spatiotemporal hemodynamic responses measured with fNIRS and fMRI data. Recently, Klein et al.^44^ performed an fMRI-based validation study of asynchronous CW-fNIRS of SMA recordings during MA and MI in the elderly population. Highly comparable correspondence was achieved during MI and MA, with both HbO and HbR presenting high spatial specificity in the latter when compared with fMRI. Complementary to these results, we provide a new approach, with the upside of establishing a truly multimodal framework, that can be used in synchronous and asynchronous fNIRS/fMRI recording (guaranteeing the same experimental protocol and timing), to locate cortical activations in fMRI space, on an individual and group-level, using corresponding fNIRS concentration data, for a motor coverage.

This study’s relatively small sample size, limited by the number of participants that previously underwent the (f)MRI acquisition, hampers the generalization of our findings to the general population. Future studies need to be performed to validate this approach using other paradigms to locate other cortical regions. Furthermore, the experimental design was not optimized for fNIRS, with the absence of a variable interstimulus interval (ISI) and no external recordings were performed to control for hand movement. Additionally, the use of asynchronous recordings may influence intra-subject analysis due to task performance variations. Ultimately, these variations can also influence signal profiles and reduce the general same-subject similarity of these two signals. Using a single-channel approach (as an alternative to p.e, simple/weighted average of multiple channels) serves as a more demanding feasibility test for this framework. In addition, using GLM results per chromophore and not a comparison between the same channel allows for maximizing the differences between possible correspondence between fNIRS measurements and fMRI BOLD signal, without any bias towards the chromophore from which the channel was selected. Other channel selection approaches could have been proposed (p.e, data-driven approaches), however we opted for use of the same hypothesis-driven approach for fMRI-ROI selection and channel selection (GLM). Future studies could include comparing different channel selection techniques as well as different fMRI ROI definition.

## Conclusion

Our study contributes to the understanding of the spatial correspondence between fNIRS and fMRI hemodynamic response measurements. Through a multimodal approach, using subject-specific fNIRS-based cortical signals as predictors of interest, we were able to identify activation in motor-related regions clusters in asynchronously recorded corresponding fMRI data. Consistent group-level activation was found in areas of the motor network, similar to corresponding fMRI and fNIRS-based group-level activation patterns, independently of the chromophore used. Similarly, no significant difference was found in individual-level peak activation and the proportion of active clusters within the fMRI functionally defined M1 and PMC region using oxy, deoxy, and total-hemoglobin data from channels more active during MA and MI, respectively. Ultimately, these findings infer the possibility of translating spatial neuronal activity information from fMRI motor paradigms into an fNIRS setup with oxy and deoxy-hemoglobin concentration, guaranteeing proper data quality and validity of measurements to uncover motor function.

## Data Availability

Data and materials may be available upon request to the corresponding author subject to transfer agreements.
